# Coral Productivity Is Co-Limited by Bicarbonate and Ammonium Availability

**DOI:** 10.3390/microorganisms8050640

**Published:** 2020-04-28

**Authors:** Stephane Roberty, Eric Béraud, Renaud Grover, Christine Ferrier-Pagès

**Affiliations:** Centre Scientifique de Monaco, 98000 Monaco, Monaco

**Keywords:** carbon limitation, nitrogen limitation, photosynthesis, calcification, Symbiodiniaceae

## Abstract

The nitrogen environment and nitrogen status of reef-building coral endosymbionts is one of the important factors determining the optimal assimilation of phototrophic carbon and hence the growth of the holobiont. However, the impact of inorganic nutrient availability on the photosynthesis and physiological state of the coral holobiont is partly understood. This study aimed to determine if photosynthesis of the endosymbionts associated with the coral *Stylophora pistillata* and the overall growth of the holobiont were limited by the availability of dissolved inorganic carbon and nitrogen in seawater. For this purpose, colonies were incubated in absence or presence of 4 µM ammonium and/or 6 mM bicarbonate. Photosynthetic performances, pigments content, endosymbionts density and growth rate of the coral colonies were monitored for 3 weeks. Positive effects were observed on coral physiology with the supplementation of one or the other nutrient, but the most important changes were observed when both nutrients were provided. The increased availability of DIC and NH_4_^+^ significantly improved the photosynthetic efficiency and capacity of endosymbionts, in turn enhancing the host calcification rate. Overall, these results suggest that *in hospite* symbionts are co-limited by nitrogen and carbon availability for an optimal photosynthesis.

## 1. Introduction

Symbiotic associations between animals and photosynthetic microorganisms have resulted in some of the best physiological adaptations that have evolved in the animal kingdom [1]. Such associations have indeed increased fitness and aptitude for food acquisition, related to the photosynthetic capacity of the autotrophic symbionts and the capture of external prey by the host. The symbiosis established between reef-building corals (Cnidaria; Anthozoa; Scleractinia) and dinoflagellates of the Symbiodiniaceae family [2,3], is one of the most well-known marine nutritional symbioses, because corals are the cornerstone of reef ecosystems, providing, through their calcification process, a tridimensional structural framework that support a high biodiversity and productivity [4]. Their ecological success in nutrient-poor tropical waters is due to the sharing and the tight recycling of nutrients between the partners [5,6]. Symbionts, through photosynthesis, transform dissolved inorganic carbon (DIC) and nitrogen (DIN), as well as other nutrients, into organic molecules which are translocated to the host for most of its metabolic needs, such as respiration, calcification and energetic reserves [7,8,9]. In turn, the animal provides its symbionts with nutrients coming from the environment and from its metabolic waste products [10,11,12].

An efficient acquisition of DIC by the symbionts is a key process for the health of the symbiotic association, because the amount of DIC fixed and assimilated by the symbionts will also determine the amount of photosynthates transferred to the host for its own needs [9]. Both partners have developed multiple ways of acquiring and concentrating carbon for photosynthesis. In addition to CO_2_ derived from respiration, the animal delivers DIC from the water column (mainly HCO_3_^−^) to the symbionts through a combination of external and intracellular carbonic anhydrases [13,14,15], specific transporters [16] and H^+^-ATPase [17]. Then the CO_2_ concentrating mechanism (CCM) of the algae takes over to maximize carbon fixation [18,19]. Even though oceanic concentrations of DIC are high, DIC can be a source of limitation for the primary production of the symbionts [15,20,21] although not in all coral species or environmental conditions [15,22]. This is due to the fact that the adequacy of the supply of DIC to the symbionts (with respect to saturation of demand) depends on diverse environmental factors, but also on symbiont density or holobiont metabolic needs, and is not yet well understood. Since symbiotic carbon acquisition is a key process for coral health, conditions which lead to increased photosynthetic efficiency of the symbionts under varying environmental conditions are therefore of interest for reef biologists, especially in this era of global changes.

The nitrogen environment and nitrogen status of the coral symbionts is one of the important factors determining optimal photosynthetic carbon acquisition and hence holobiont growth. In plants and free-living algae, carbon fixation and nitrogen assimilation are interdependent, because fixed carbon need to be coupled to a nitrogen source for amino acid production [23,24,25,26,27]. For instance, it was shown that CO_2_ deprivation inhibits NH_4_^+^ uptake in the unicellular alga *Cyanidium caldarium* [24], but also that internal nitrogen levels modulate the photosynthetic response of the organisms to DIC enrichment [28]. In the coral-dinoflagellate association, the carbon-nitrogen coupling has been less studied than in plants and algae and has led to opposite conclusions, also depending on the nitrogen source (ammonium versus nitrate) and the nitrogen to phosphorus (N:P) ratios considered [29,30]. Overall, the above studies showed negative impacts of increased N:P ratios and nitrate enrichment on coral photosynthesis and health and a positive effect of ammonium enrichment. Nevertheless, for the same nitrogen source such as ammonium, controversies still exist. On the one hand, overall carbon acquisition (per holobiont surface area) or per symbiont cell was enhanced by heterotrophic feeding (ammonium source) or direct ammonium supplementation, suggesting that nitrogen, not carbon, was limiting symbiont photosynthesis [31,32,33]. On the other hand, when nitrogen enrichment of the seawater increased symbiont density [34,35,36,37], symbionts may compete for CO_2_ acquisition, and present decreased rates of photosynthesis per symbiont cell [34,36]. Such discrepancy in the effect of nitrogen enrichment on carbon acquisition can be due to differences in DIC limitation of the symbionts but remains to be investigated.

All together, these results suggest that the role of inorganic nutrient availability on the photosynthetic capacities of the coral holobiont is partly understood. The aim of this study was to test the DIC and ammonium (NH_4_^+^) limitation for the photosynthesis of the symbionts associated to the scleractinian coral *Stylophora pistillata*. We first assessed whether symbionts of *S. pistillata*, were DIC or NH_4_^+^ limited under our oligotrophic culture conditions, by enriching seawater with either 4 µM ammonium or 6 mM bicarbonate. We also tested DIC limitation under nitrogen-enriched conditions. Such conditions will occur more and more frequently in eutrophic coastal reef waters, following urban and agricultural development [30,38,39]. In the present context of global change and anthropogenic impacts on coastal waters, experimental studies like this one will help develop adaptive management strategies for the future of coral reefs.

## 2. Materials and Methods

### 2.1. Biological Material and Experimental Settings

The effects of ammonium and bicarbonate enrichment were assessed in colonies of the scleractinian coral *S. pistillata* originating from the Gulf of Aqaba (Red Sea, Jordan). These colonies host Symbiodiniaceae of the genus *Symbiodinium* (ITS2 type: A1) and were grown at 25 °C, under a 12:12h light/dark cycle with 400 W metal halide lamps, providing about 200 µmol photon m^−2^·s^−1^ at the level of the colonies (measured with a spherical quantum sensor (LI-193) connected to a LI-COR data logger (LI-1000, LI-COR Biosciences, Lincoln, NE, USA). Seawater in the aquaria was continuously renewed by natural oligotrophic seawater and colonies were fed twice a week with freshly hatched *Artemia salina* nauplii. A total of 264 coral nubbins of similar size were cut from 6 parent colonies (22 per parent colony), suspended by nylon fishing lines and were evenly and equally distributed in eight aquaria of 25 L. Nubbins were let to recover for 5 weeks, under the same conditions as the parent colonies except for light intensity that was increased to 250–300 µmol photon m^−2^s^−1^. Feeding was stopped 2 weeks prior to the beginning of the experimental treatments to avoid any interaction with the addition of inorganic nitrogen. After this period, each experimental treatment ran for 21 days and coral nubbins were sampled at days 1, 3, 7, 11 and 21. The 4 experimental treatments were tested in duplicated aquaria and consisted of: ambient ammonium (<0.3 µM) and bicarbonate (2 mM) concentrations (control condition); 4 µM ammonium and ambient bicarbonate concentration (‘+iN’ condition); ambient ammonium concentration and 6 mM bicarbonate (‘+iC’ condition); 4 µM ammonium and 6 mM bicarbonate (‘+iC and iN’ condition) (see Table 1). Stock solutions of seawater enriched with 5 mM ammonium and/or 66.7 mM bicarbonate were prepared in 100 L tanks (pH adjusted to 8.2) and injected into the respective experimental aquaria with a peristaltic pump, at a constant flow rate of 3.75 mL min^−1^. The ammonium concentration chosen is high but reflects the concentrations that may occur in some eutrophicated coral reefs [39]. Conversely, even if the predicted increase in atmospheric CO_2_ and consequent acidification of the oceans will increase the abundance of HCO_3_^−^ ions and dissolved CO_2_ at the expense of CO_3_^2−^ [40], the HCO_3_^−^ concentrations to which coral colonies were exposed in this study are higher, but comparable to previous studies which have investigated the limitation of symbionts in iC [20,41].

### 2.2. Water Chemistry

During the experiment, ammonium concentration, total alkalinity, and pH (NBS scale) in each treatment were monitored on a regular basis. Ammonium concentration was determined with a Proxima autoanalyzer (Alliance Instrument, Frépillon, France), according to the method of Holmes, et al. [42]. Total alkalinity was measured using a 888 Titrando titrator (Metrohm, Herisau, Switzerland). Seawater carbonate chemistry measurements (Table 1) was calculated using the program CO2SYS developed by [43], with the following constants: K1, K2 from [44]; KSO_4_ source from [45]; and total boron source from [46].

### 2.3. Symbiodiniaceae Density and Chlorophylls Content

In order to determine *Symbiodinium* density, coral tissue was separated from the exoskeleton with compressed air in a known volume of filtered seawater (FSW). The slurry produced was homogenized using a Potter tissue grinder and the final volume of the homogenate was recorded. Three 125 µL sub-samples was taken to measure symbiont cell concentration using a Z1 Beckman Coulter Particle Counter (Beckman, Brea, CA, USA). Chlorophylls *a* and *c_2_* were extracted from 2 mL tissue homogenate in 2 mL 100% methanol at 4 °C. After debris removal (centrifugation at 16,000× *g* for 20 min at 4 °C), chlorophyll *a* and *c_2_* concentrations were determined by spectrophotometry according to the equations of [47] for dinophytes. Data were then normalized to the surface area of the coral nubbins, measured using the wax dipping method described in [48].

### 2.4. Pigment Content Analysis

Six coral nubbins were sampled in each experimental condition, after 1, 3, 7, 11 and 21 days. Samples were placed for 15 min under 400 µmol photons m^−2^ s^−1^ before being flash frozen in liquid nitrogen. They were then lyophilized, and pigments were extracted in 1.5 mL of MeOH (HPLC Grade, Merck, Darmstadt, Germany). Samples were then vortexed for 3 × 15 s with 0.5 mL glass beads (710–1180 µm; Sigma Aldrich, St. Louis, MO, USA) before being centrifuged at 16,000 × g for 20 min at 4 °C. Pigments were then separated by reverse-phase HPLC, using Shimadzu Prominence HPLC system, comprising a DGU-20A5R Degassing Unit, a LC-20AT Liquid chromatograph, a SIL-20AC Autosampler, a CTO-10ASVP Column Oven and a SPD-M20A Diode Array Detector (Shimadzu, Kyoto, Japan). The HPLC column (Nova Pak C18, 60A column, 150 mm length and 4 µm pore size) was eluted with a mobile phase gradient (1 mL min^−1^) set to the program described in [49]. Absorbance chromatograms were detected at 430 nm and quantified with pigments standards purchased from DHI Lab (Horstholm, Denmark). Acquisition and data treatment were performed using the Waters Empower software (Waters, Milford, MA, USA).

### 2.5. Chlorophyll Fluorescence Measurements

Chlorophyll fluorescence emission was measured using a DUAL-PAM 100 (Fiber version; Walz, Effeltrich, Germany), with the fiber optic fitted to a temperature-controlled chamber (50 mL volume; 25 °C). After a dark adaptation (DA) of 15 min, a light saturation curve comprising six irradiance steps (0, 75, 150, 300, 600 and 900 µmol photon m^−2^ s^−1^; measured with a Submersible Spherical Micro Quantum Sensor (Walz) connected to a LI−250A light meter (Li−Cor)) of 5 min each was conducted, with gain (G) and damping (D) set to 5 and 1 ms, respectively. The minimal fluorescence levels (F_0_ in the dark and F_S_ in the light) were determined by applying weak modulated pulses of red measuring light (ML = 10). A 0.8 s saturating pulse of actinic light (SI = 10 corresponding to an intensity of ≈4000 µmol photon m^−2^ s^−1^) was then applied at the end of each step to measure the maximum fluorescence level (F_M_; F_M_’ in the light). The maximum photochemical quantum yield of PSII (F_V_/F_M_) was calculated at the end of the dark incubation as (F_M_−F_0_)/F_M_. The effective quantum yield of PSII (ɸPSII) was calculated at the end of each light step as (F_M_’−F_S_)/F_M_’. The non-photochemical quenching (NPQ) was evaluated as (F_M_−F_M_’)/F_M_’ and the relative electron transport rates (µmol e^−^ m^−2^ s^−1^) through PSII (rETR_PSII_) were calculated as the product of actinic light intensity by the corresponding ɸPSII.

The relative flux of electrons originating from PSII that reduce O_2_ through the action of the oxygen-dependent chloroplastic alternative electron flows (Mehler reaction, chlororespiration, photorespiration…) has been estimated according to the methodology of [50] with slight modifications. Briefly, the O_2_-dependent rETR at the induction of photosynthesis was calculated as the difference between rETR_PSII_ values in the presence and absence of O_2_. The rETR_PSII_ was determined during the induction phase of photosynthesis and was measured at the end of an illuminated period of 15 s at an intensity of 340 µmol photons m^−2^ s^−1^. The O_2_ depletion in the samples was obtained in 5 min using 50 mM glucose, 200 U mL^−1^ glucose oxidase and 200 U mL^−1^ catalase. 

### 2.6. Oxygen Exchange Measurements

Oxygen exchange rates were measured using a Clark-type electrode connected to a Strathkelvin 928 Oxygen System (Strathkelvin Instruments Ltd., North Lanarkshire, UK) and fitted to a temperature-controlled chamber (50 mL volume; 25 °C). The oxygen electrode was calibrated against air-saturated (100% oxygen saturation) and sodium dithionite saturated (0% oxygen saturation) seawater. Actinic light was provided by LED light sources (LEDS-5MP/WS, Monacor, Bremen, Germany; color temperature of 5500 K). Values at steady state were collected after 5 min illumination at each light intensity (as described for chl fluorescence measurements). Data were normalized to the surface area of the coral nubbins or to the endosymbiont concentration.

### 2.7. Calcification and Ammonium Uptake Rates

Calcification rates were measured on six coral nubbins in each treatment using the buoyant weight technique [51]. All results were normalized to skeletal surface area and expressed in % day^−1^. The uptake rates of ammonium were determined by measuring the depletion of ammonium over time in ammonium-enriched beakers, 1, 3, 7, 11 and 21 days after the beginning of the experiment. Six coral nubbins per conditions were introduced in 250 mL beakers containing 200 mL FSW and a magnetic stirrer to ensure a complete homogenization of the medium. A beaker without coral served as a control for changes in ammonium concentrations that could be due to non-coral specific uptake. The beakers were then partially immerged in a water bath at 25 °C and exposed to a light intensity of 350 µmol photon m^−2^ s^−1^. After 30 min of acclimatization, an enriched FSW solution containing NH_4_Cl was added to each beaker to reach an initial concentration of 5 µM NH_4_^+^. Then, 10 mL of water were sampled in each beaker immediately after enrichment and then every 10 min for 30 min. Water was filtered through a 0.45 µm syringe filter and ammonium concentration was determined with a Proxima autoanalyser (Alliance Instrument) and according to the method described in [42]. Uptake rates of ammonium were calculated as the difference between the initial and final concentrations, taking into account the diminution of the beaker volume, divided by the time, and standardized to coral surface area.

### 2.8. Statistical Analysis

Statistical analyses of the data were performed in SigmaPlot 11.0 (Systat Software, San José, CA, USA) or in Statistica 10 (StatSoft, Tulsa, OK USA). Principal component analyses (PCA) were performed on physiological parameters related to the holobiont (*Symbiodinium* density, ammonium uptake, respiration, Pmax and P/R) or to the symbiont (chlorophylls content, F_V_/F_M_, rETR_PSII_ max, NPQ max and O_2_-dependent rETR_PSII_) after log(x+1) transformation. Then, the ‘time’, ‘treatment’ and ‘time x treatment’ effects were examined by two-way repeated measures ANOVA (2W-rmANOVA). When significant differences were obtained for the ‘time x treatment’ effect, the analysis was followed by the SNK’s method for all pairwise multiple comparison. Prior to this analysis, the normality of data was confirmed with the Shapiro–Wilk test and data were arcsine square root transformed when required. The Mauchly’s Test was used to assess the sphericity assumption (independency of the repeated measures), and F-value was adjusted by a Greenhouse-Geisser correction if the assumption was not fulfilled. Differences were considered statistically significant when *P* < 0.05. All data were expressed as mean ± SD. As we did not observe any significant variation for the physiological parameters investigated at days 1 and 3, and for more clarity, we only displayed data for days 7, 11 and 21 (see Sable S1 for values at the beginning of the experiment).

## 3. Results

**Figure 1:**
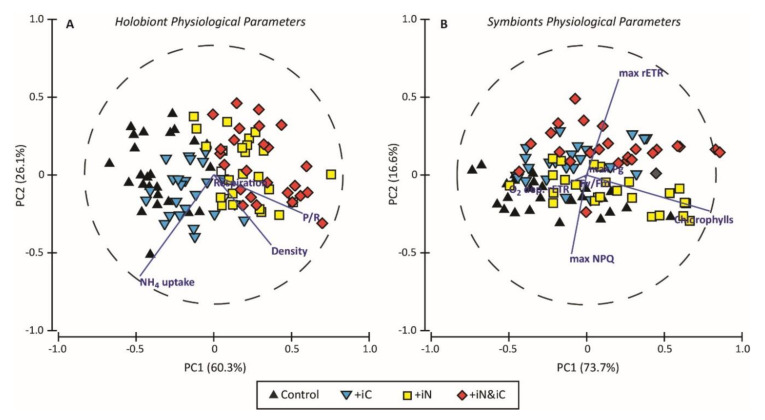
The principal component analyses bi-plots on parameters related to (**A**) the holobiont physiology and to (**B**) the symbiont physiology and photobiology. Black triangles, blue inverted triangles, yellow squares and red diamonds represent, respectively, *S. pistillata* nubbins exposed for 3 weeks to natural seawater (control) and to seawater enriched with 6 mM HCO_3_^−^ (+iC), 4 µM NH_4_^+^ (+iN), both (+iC & iN). Blue lines represent the physiological traits used as descriptors.

PCA analyses were performed to compare the physiological traits of the corals and their endosymbionts maintained under the four conditions (Figure 1). These analyses showed a clear separation in the distribution of the data related to the holobiont physiology (Figure 1A), with the PC1 and PC2 explaining 60.3 and 26.1% of the total variance, respectively. The first axis (PC1) is mainly defined by the parameters related to photosynthesis-respiration ratio and to a lesser extent to the endosymbiont density. It clearly separates nitrogen-enriched conditions (+iC and +iN & iC) from the two other treatments. The second component (PC2) is mainly correlated (*r* = −0.784) with the NH_4_^+^ uptake parameter, and again separates nitrogen-enriched conditions from the +IC condition. For the endosymbionts’ physiological parameters (Figure 1B), the data are less clearly separated between treatments. The first two components (PC1 = 73.7% and PC2 = 16.6%) explain 93.3% of the variance. PC1 is associated with chlorophylls concentrations (*r* = 0.951) and PC2 with photosynthetic performances (max rETR, *r* = 0.741; max NPQ, *r* = −0.608).

### 3.1. Effects of Increased Ammonium Supply Compared to Control Condition.

**Figure 2:**
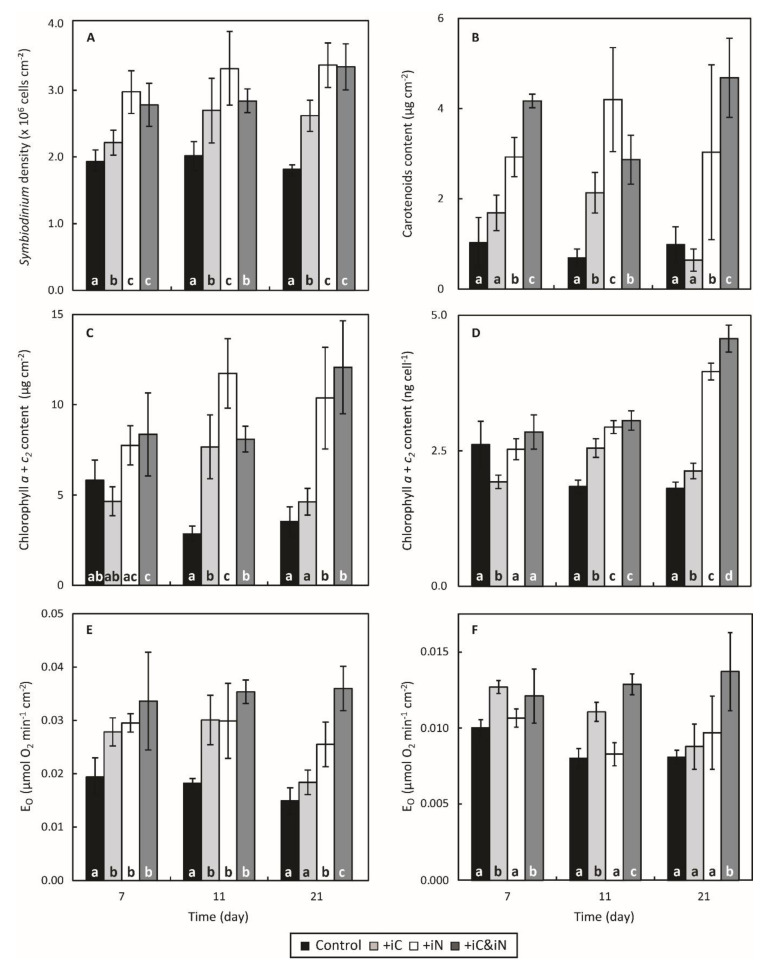
Evolution of the physiological parameters of the coral *S. pistillata* exposed for 3 weeks to natural seawater (control) and to seawater enriched with 6 mM HCO_3_^−^ (+iC) or 4 µM NH_4_^+^ (+iN) or both (+iC & iN). (**A**) *Symbiodinium* densities (× 10^6^ cells^−1^ cm^−2^), (**B**) total carotenoids concentrations (peridinin + xanthophylls + β-carotene; µg cm^−2^), (**C**) total chlorophylls concentrations (*a*+*c_2_*; µg cm^−2^), (**D**) Total chlorophylls cellular concentrations (*a+c_2_*; ng cell^−1^), (**E**) gross photosynthesis of the holobiont (gross O_2_ evolution by PSII (E_O_); µmol O_2_ min^−1^ cm^−2^) measured at 600 µmol photons m^−2^ s^−1^, (**F**) gross photosynthesis of the symbionts (fmol O_2_ min^−1^ cell^−2^). Data are presented as mean ± SD (*n* = 6). Different letters indicate statistically significant differences between each treatment for one time point (*P* < 0.05).

**Figure 3:**
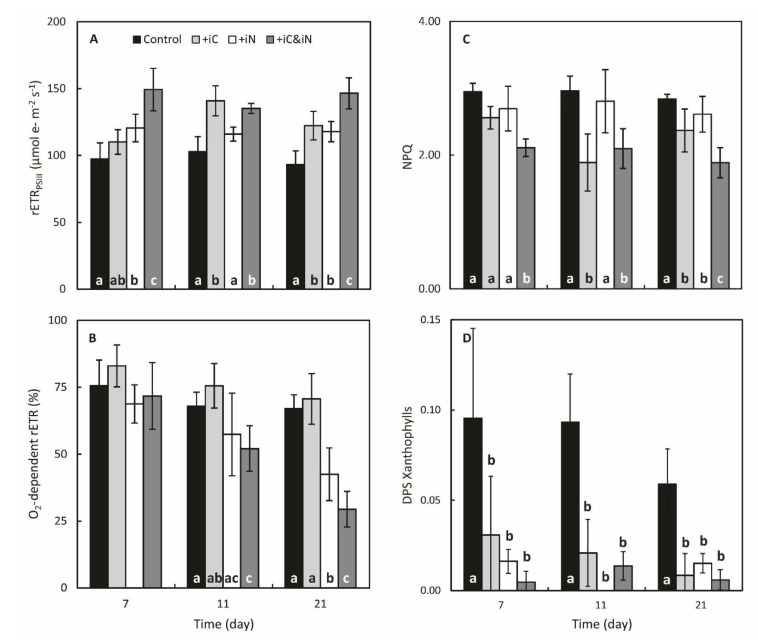
Evolution of the photosynthetic parameters of the endosymbionts of *S. pistillata* exposed for 3 weeks to natural seawater (control) and to seawater enriched with 6 mM HCO_3_^−^ (+iC) or 4 µM NH_4_^+^ (+iN) or both (+iC & iN). (**A**) the relative electron transport rate through PSII (rETR_PSII_; µmol e^−^ m^−2^ s^−1^) at 600 µmol photons m^−2^ s^−1^, (**B**) the O_2_-dependent rETR at the induction of photosynthesis (%), (**C**) the non-photochemical quenching (NPQ) at 600 µmol photons m^−2^ s^−1^ and, (**D**) the de-epoxidation state of the xanthophylls cycle (DPS; was measured after 15 min of illumination with 400 µmol photons m^−2^ s^−1^ as the ratio of Diatoxanthin (Dtx) to Diadinoxanthin (Ddx) + Dtx. Data are presented as mean ± SD (*n* = 6). Different letters indicate statistically significant differences between each treatment for one time point (*P* < 0.05).

The enrichment of seawater with 4 µM NH_4_^+^ alone (measured concentrations of 4.36 ± 1.23 µM; Table 1) significantly affected several physiological parameters of *S. pistillata* and its endosymbionts compared to the control condition. The *Symbiodinium* density was higher at day 7 (*P* < 0.05) and seemed to have reached a steady-state density with 3.32 and 3.38 × 10^6^ cells cm^−2^ (154 and 164% of the control) after 11 and 21 days of exposure to this treatment, respectively (Figure 2A). This higher *Symbiodinium* density coincided with an increase in chlorophyll and carotenoid content per endosymbionts and per skeletal surface area (Figure 2B,D and Figure S2). The total chlorophyll and carotenoid contents were higher than the controls after day 11 (Figure 2C,D; *P* < 0.05) and day 7 (Figure 2B and Figure S2; *P* < 0.05), respectively, and remained higher until the end of the experiment. These statistical differences could be partly explained by the decrease in the total chlorophyll content in control corals after day 7 (Figure 2C,D). The Chl-*a*/Chl-*c_2_* ratio was not affected in the NH_4_^+^-enriched condition and was around two Chl-*a* molecules per one Chl-*c_2_* molecule (Figure S3). However, after 3 weeks of treatment, the proportion of Chl-*c_2_* was slightly increased in the control condition and hence the ratio was significantly lower (*P* < 0.001). As a result of the increase in *Symbiodinium* density and in photosynthetic pigments content from day 7 to day 21, the maximum gross photosynthesis cm^−2^ was enhanced between 58 and 73% comparatively to the controls (Figure 2E; *P* < 0.001). While the values of maximum gross photosynthesis cell^−1^ (Figure 2F) maximal photochemical quantum yield of PSII (F_V_/F_M_; Figure S4) and non-photochemical quenching (NPQ; Figure 3C) remained relatively close to controls throughout the experiment, at day 21 the relative electron transport rate through PSII (rETR_PSII_) was increased by 26% (*P* = 0.002) and the O_2_-dependent rETR was 1.6 times lower (*P* < 0.001; Figure 3A,B). Calcification rate of corals supplied with ammonium increased significantly (*P* = 0.005), on average by 23% (Figure S5).

### 3.2. Effects of the Increased Bicarbonate Supply Compared to Control Condition

The increased availability in bicarbonate alone (6 vs. 2 mM; Table 1) in the iC treatment positively impacted several physiological parameters of the holobiont compared to the control condition. *Symbiodinium* density was slightly higher at day 7 (*P* = 0.048) and increased up to a density of 2.62 ± 0.23 × 10^6^ cells cm^−2^ at day 21 (144% increase; *P* < 0.001; Figure 2A). Rates of gross photosynthesis expressed per surface area differed significantly from the controls at day 7 and 11 (*P* = 0.003, *P* < 0.001) and increased to 0.028 ± 0.003 and 0.030 ± 0.005 µmol O_2_ min^−1^ cm^−2^ (147 and 166% increase), respectively (Figure 2E; similar observations can be made on rates of gross photosynthesis when expressed per cell (Figure 2F)). Likewise, rETR_PSII_ measured at 600 µmol photons m^−2^ s^−1^ increased significantly by about one third after day 11 (*P* < 0.001; Figure 3A). NPQ declined strongly at day 11 (*P* < 0.001; Figure 3C) but the difference of the means between controls and treated samples at day 21 was lower but yet significant (*P* = 0.031). The NPQ decline is consistent with the lower de-epoxidation state of the xanthophylls observed from day 7 (*P* < 0.05; Figure 3D). Corals also calcified more in this condition compared to the controls (av. 34%; *P* < 0.001; Figure S5).

### 3.3. Effects of the Combined Supply of Ammonium and Bicarbonate Compared to All Conditions

**Figure 4:**
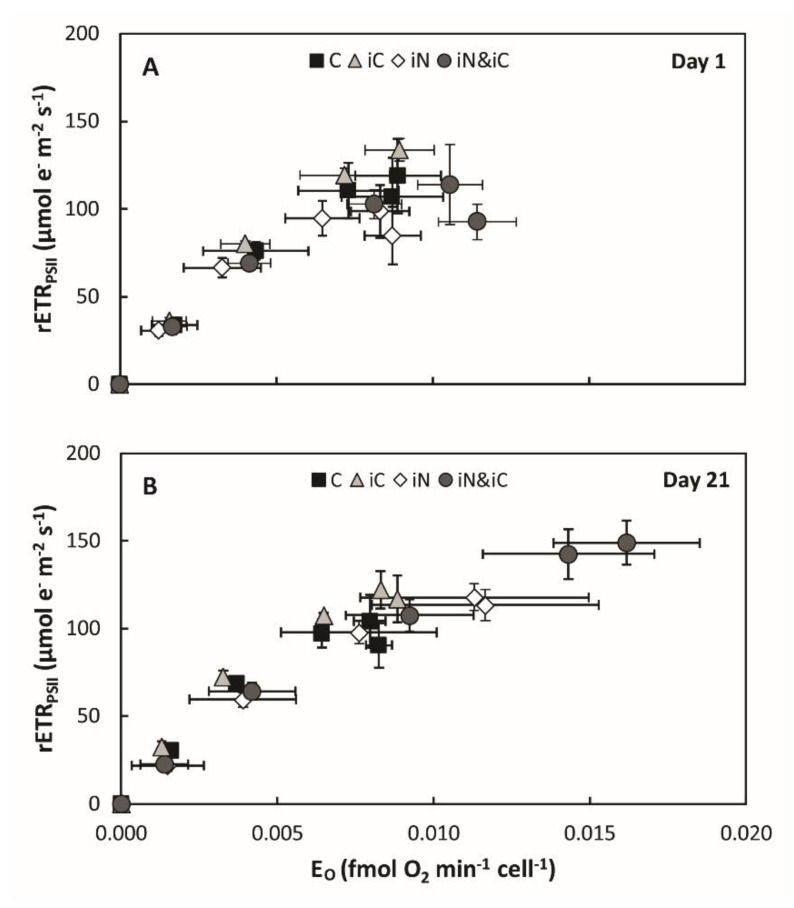
Comparison of the relative electron transport rate through PSII (rETR_PSII_; μmol e^−^ m^−2^ s^−1^) and gross O_2_ evolution by PSII (E_O_; fmol O_2_ min^−1^ cell^−1^) at steady-state photosynthesis in *S. pistillata*, after 1 day (**A**) and 3 weeks (**B**) of exposure to natural seawater (control) and to seawater enriched with 6 mM HCO_3_^−^ (+iC) or 4 µM NH_4_^+^ (+iN) or both (+iC & iN). Measurements were conducted at 0, 70, 150, 300, 600 and 900 μmol photons m^−2^·s^−1^ and data are presented as mean ± SD (*n* = 6).

The enrichment of seawater with both 6 mM HCO_3_^−^ and 4 µM of NH_4_^+^ resulted in a 40% increase of the *Symbiodinium* density, at day 7 and 11 (*P* = 0.002 and *P* < 0.001, respectively), and 85% on average at day 21 (*P* < 0.001). On the last day of the experiment, the average density in this treatment was higher than in the samples of the iC treatment alone but was similar to that of the iN treatment (*P* < 0.001; Figure 2A). The total chlorophylls and carotenoids content expressed per coral surface (Figure 2B,C) or symbiont cell (Figure 2D and Figure S2) significantly increased throughout the experiment. After 21 days of treatment, Chl *a*+*c_2_* reached a concentration of 4.57 ± 0.25 ng cell^−1^ (12.07 ± 2.58 µg cm^−2^) and was significantly higher than in all other treatments (Figure 2D; *P* < 0.001). Similarly to the iN treatment, the Chl-*a*/Chl-*c_2_* ratio remained stable during the experiment but significantly higher than the other treatments (*P* < 0.05; Figure S3). Globally, corals nubbins in the iC & iN treatment were also those that had the highest photosynthetic activity with: a maximum gross photosynthesis per symbiont cell that were respectively 1.7, 1.6 and 1.4 times higher than the control, iC and iN treatments, respectively (*P* < 0.05; Figure 2E,F); a relative electron transport rate through PSII 25 to 50% quicker on average (*P* < 0.001; Figure 3A); a NPQ significantly reduced by 20 to 30% (*P* < 0.001; Figure 3C); and a de-epoxidation state of the xanthophylls (Figure 3D) an order of magnitude lower than the control (*P* < 0.001; Figure 3D) but similar to the two other treatments. The O_2_-dependent rETR at the induction of photosynthesis decreased significantly from 72 ± 12% at day 7 to 29 ± 7% at day 21 in samples enriched with both ammonium and bicarbonate compared to control, iC, and iN samples (*P* < 0.05; Figure 3B). Figure 4 illustrates photosynthesis-irradiance curves where rETR_PSII_ was evaluated in parallel with gross O_2_ evolution by PSII (E_O_) at steady state of photosynthesis. This method has been successfully applied in the past to evaluate the magnitude of oxygen-dependent chloroplastic alternative electron flows (AEF; e.g., Mehler reaction, chlororespiration, photorespiration…) in various species of Symbiodiniaceae isolated in culture [52,53]. In the present study, rETR_PSII_ and E_O_ both saturated at light intensities above 300 µmol photons m^−2^·s^−1^ and 600 µmol photons m^−2^ ·s^−1^ at the beginning of the experiment and after 3 weeks of bicarbonate and ammonium supplementation, respectively (Figure S6). However the shape of the relationship between both parameters (i.e., linear) differed from those previously reported in [53] and more recently in [52] (i.e., non-linear with net VO_2_ saturating more rapidly than rETR_PSII_). Finally, the calcification rate measured in the iC & iN condition was also higher than in the control condition (*P* = 0.006; Figure S5) but similar to the rates measured in the other experimental treatments.

## 4. Discussion

This study reports the first assessment of a combined inorganic carbon (HCO_3_^−^) and nitrogen (NH_4_^+^) enrichment on the physiological traits of a coral-dinoflagellate symbiosis. Although positive effects on coral physiology could already be observed with the supplementation of one or the other nutrient, the most important and positive changes were observed when both nutrients were provided, due to an interactive relationship between NH_4_^+^ and DIC acquisition. These results suggest that coral symbionts are co-limited in nitrogen and carbon for an optimal photosynthesis.

### 4.1. The Increased Availability of HCO_3_^−^ and NH_4_^+^ Improves Photosynthetic Processes in Endosymbionts

The increased availability in bicarbonate or ammonium alone had significant, but time-dependent effects on symbiont physiology. Since symbionts are generally nitrogen limited *in hospite* [54], ammonium supplementation induced a fast response of the symbionts, which showed a significant increase in growth and pigment content as well as an increase in some of their photosynthetic processes (E_O_ per symbiont and rETR) after only 7 days of incubation. Conversely, the rate at which iC supplementation affected symbiont physiology was dependent on the parameter considered. Nevertheless, after 21 days, the increased availability in either bicarbonate or ammonium improved significantly the photosynthetic efficiency (rETR_PSII_) and/or capacity (O_2_ production per symbiont cell) of the endosymbionts of *S. pistillata* (Figure 3A and Figure 2F), partly due to a significant increase in chlorophylls content per cell. This suggests that the activity of the photosynthetic apparatus is impacted by the nitrogen availability and that the *in hospite* symbionts are CO_2_ limited. The positive effect of bicarbonate supplementation also translated into a significant decrease in NPQ and DPS xanthophylls (Figure 3). These results are indicative of lower excitation pressure on *Symbiodinium*’s photosynthetic apparatus in the enriched conditions (see below for more explanations). A DIC limitation of *in hospite* symbionts is in agreement with previous studies, which reported an increase in photosynthesis per symbiont cell or chlorophyll content [15,20,21]. Tansik et al. [15] postulated that the cause of such DIC limitation of photosynthesis might be related to the activity of the coral external carbonic anhydrase and therefore, to the host regulation of DIC delivery to endosymbionts.

The major finding of this study is the co-limitation of symbiont photosynthesis by inorganic nitrogen and carbon. Indeed, several symbiont parameters, such as the chl and carotenoid content per cell, the rates of oxygen production per cell and the rETR were significantly higher under the double iC & iN enrichment compared to all other conditions after 21 days of incubation (Figure 2D,F, Figure S2 and Figure 3A). Concomitantly, symbionts showed the highest decrease in NPQ (ca. 30% Figure 3C) and a constant low de-epoxidation state of the xanthophylls compared to control (Figure 3D). These results suggest that the excitation pressure on *Symbiodinium*’s photosynthetic apparatus in the enriched conditions is lower and more energy is allocated to photochemistry. The 50% reduction in the O_2_-dependent rETR at the induction of photosynthesis (Figure 3B) comes in support to this hypothesis. Indeed, the O_2_-dependent rETR is a parameter estimating the amplitude of AEF involving O_2_ as the final electron acceptor, and has been demonstrated to be related to the Mehler reaction (i.e., electrons originating from PSII that reduce O_2_ at the PSI acceptor side) in Symbiodiniaceae [30]. As the Mehler-reaction acts as a photoprotective mechanism, a decrease of O_2_-dependent rETR is indicative of an increased availability of electron acceptors downstream PSI. The evolution of the relationship between rETR_PSII_ and the O_2_ net exchange rates during steady state photosynthesis (Figure 4) is also consistent with the above results. Such relationship became more linear at the highest light intensities (> 300 µmol photons m^−2^·s^−1^) after 3 weeks of the double iC & iN enrichment suggesting that coral symbionts have a more efficient light phase, very likely because more electron acceptors downstream PSI are available at the induction of photosynthesis and during steady state. It also suggests that rapid variations in light intensity (e.g., underwater light flecks) and the excess energy that co-occurs, could be more easily withstood by the photosynthetic apparatus of the endosymbionts.

The coupling between photosynthesis and nitrogen metabolism is well-known in microalgae [55]. Since N is involved in the composition of photosynthetic pigments like chlorophylls and phycobilins, N limitation was shown to change the ratio of chlorophyll-*a* to accessory pigments, to cause a reduction in thylakoid absorptivity, to negatively impact photosynthetic enzymes such as Rubisco, as well as the efficiency of energy transfer through the photosynthetic chain (reviewed in [56]). In corals, once ammonium is absorbed by the endosymbionts, it is assimilated into glutamate and glutamine thanks to the glutamine synthetase (GS)/glutamate synthase (GOGAT) cycle [57]. This metabolic pathway is driven by energy and reducing power derived from photosynthesis (reduced Ferredoxin, ATP and NAD(P)H)) and the synthesized products are the precursors for the biosynthesis of major N compounds (amino acids, nucleic acids, chlorophylls, secondary metabolites…) [58]. All together these results suggest that the increased availability of both HCO_3_^−^ and NH_4_^+^ improves the photosynthetic efficiency and capacity by providing more electron sinks through the Calvin-Benson cycle and the ammonium assimilation pathway. Moreover, by positive feedback, the resulting higher photosynthetic activity provides more carbon molecules onto which nitrogen can be fixed and thus impacts positively the symbiont biomasses.

### 4.2. The Increased Availability of HCO_3_^−^ and NH_4_^+^ Sustains the Growth of the Holobiont

When expressed per unit of coral surface area, gross photosynthesis rates, which are now a proxy of the total amount of photosynthetic carbon acquired by the holobiont, were increased in all enriched conditions (Figure 2E). Such a response at the holobiont level has been previously reported on *S. pistillata* when supplemented with bicarbonate [59] or ammonium [33,34,36]. In our study it is related to the increased symbiont biomass that occurred within a week. While symbiont growth limitation by nitrogen availability is relatively well-known (e.g., [33,35,37,60,61]) it has been less reported for CO_2_/HCO_3_^−^ availability, alone or in combination with iN, which is a new observation in coral studies. The stimulation of symbiont growth observed here suggests that the enhancement of photosynthesis is accompanied by a higher carbon fixation in the symbionts. A co-limitation of primary productivity by carbon and nitrogen has been well studied in terrestrial plants and macroalgae. In these models, it was demonstrated that N uptake and assimilation rates were significantly enhanced following culture at high pCO_2_ (e.g., [62,63,64]). The increased demand in mineral nutrients such as nitrogen and phosphorus is generally needed to support a higher growth rates of plants and algae under high iC availability. It has to be noticed that the uptake rates of iN observed in the present study were also always higher under the control and iC enrichments (Figure S1), suggesting higher needs in iN under these two conditions; however, since the uptake rates of iN were measured at the holobiont level (host + symbionts + microbiome), we cannot conclude about the occurrence of a similar effect in coral endosymbionts. In addition, a meta-analysis on plants demonstrated that high iC levels significantly decreased nitrogen concentrations in plant tissues by 2 to 57% depending on species, as a consequence of a combination of increase carbohydrate concentrations, starch accumulation, decreased investment in Rubisco, and changes in tissue chemical composition (reviewed in [65]). Although this remains to be tested, similar changes might have occurred in our coral symbiosis following iC enrichment, which have requested higher nitrogen acquisition by the symbionts, only possible, in oligotrophic waters, with an additional input of iN. In turn, the increased nitrogen input has promoted *Symbiodinium* proliferation within host tissues, resulting very likely in a greater competition for CO_2_/HCO_3_^−^ between individual endosymbiotic cells [66]. Therefore, when CO_2_ is provided concomitantly with nitrogen, photosynthetic rates at the symbiont and the holobiont levels are further improved.

The enhancement of areal gross photosynthesis does not necessarily result in enhanced carbon translocation into the host tissue, and thus enhanced host benefit. This is due to the fact that growing symbiont populations might turn into parasitism (keeping nutrients for their own needs) under certain conditions such as thermal stress or nutrient enrichment [67,68,69]. The impact of nutrient enrichment on the coral-algae symbiosis is however dependent on the forms (organic versus inorganic, ammonium versus nitrate) and nitrogen:phosphorus ratio [29,70]. Although nutrient supply in the form of plankton particles or ammonium, increases both the areal rates of photosynthesis and of carbon translocation in the same *S. pistillata* species than as used here [71,72], no study has investigated the effect of a double enrichment in inorganic carbon and nitrogen on the translocation rates of photosynthates. Nevertheless, the fact that calcification rates were similarly increased in all enriched treatments (either with iC, iN or iN & iC) compared to the control conditions suggest that the improvement in the photosynthetic efficiency of the symbionts, and the overall increase in the areal rates of photosynthesis benefit to the coral host. Indeed, calcification is an energy-demanding process, which cannot be achieved under sub-optimal nutritional conditions [73]. Some previous studies actually linked increased calcification rates with high bicarbonate/carbonate concentrations [20,57,74]. They explained the effect by the establishment of a high aragonite saturation state (Ω aragonite), favorable for calcification, or by a suppression of competition between calcification and photosynthesis for iC. However, the results obtained here with iN enrichment alone suggest that the enhancement of calcification in our experiment was not linked to a higher Ω aragonite, or to a direct nitrogen or carbon effect on the calcification process, but rather to higher rates of photosynthesis, which provided more energy, as well as organic molecules for the synthesis of the organic matrix of the coral skeleton [75]. As previously observed [76], the increase in calcification rate under the iC&iN enrichment was much lower than the photosynthetic increase (22% against 133%), suggesting that coral calcification was already at a sub-optimal rate under non enriched conditions or that it was limited by other factors, independent of the photosynthetic process.

Eutrophication, with excessive amount of N, P, and CO_2_, and insufficient amount of dissolved O_2_, is becoming a serious problem causing a global deterioration of reef environments [77,78]. Our results however suggest that there is an interactive effect of high iC and iN on the photosynthesis and general metabolism of scleractinian corals like *S. pistillata* which will have to be further studied. In particular, it is well-known that nitrate, which is the main by-product of eutrophication, has a different effect on coral metabolism than ammonium [29,30,70,71], in particular under low phosphorus availability. Future studies should therefore aim to investigate the combined effects of all these nutrients when considered under their different forms and availabilities.

## Figures and Tables

**Table 1:** Mean carbonate chemistry parameters and NH_4_^+^ concentrations for the four treatments representing two HCO_3_^−^ (control vs. 6000 µmol kg^−1^) concentrations and two NH_4_^+^ concentrations (control vs. 4 µM); *n* = 30.

	Temp (°C)	pH (NDS scale)	TA (µmol kg^−1^)	pCO_2_ (µatm)	[CO_2_] (µmol kg^−1^)	[HCO_3_^−^] (µmol kg^−1^)	[CO_3_^2−^] (µmol kg^−1^)	Ω aragonite	xCO_2_ (ppm)	[NH_4_^+^] (µM)
Control	25.03 ± 0.04	8.04 ± 0.02	2447.82 ± 56.69	609.52 ± 29.84	16.97 ± 0.84	1988.18 ± 34.11	186.24 ± 12.09	2.89 ± 0.19	628.80 ± 30.76	0.37 ± 0.24
+iC	25.21 ± 0.03	8.07 ± 0.05	6959.37 ± 745.45	1646.94 ± 210.85	45.64 ± 5.81	5710.35 ± 557.25	578.23 ± 109.01	8.98 ± 1.69	1699.63 ± 217.67	0.40 ± 0.34
+iN	25.04 ± 0.04	8.16 ± 0.03	2310.56 ± 37.54	407.70 ± 30.98	11.35 ± 0.87	1762.06 ± 34.54	219.27 ± 12.39	3.40 ± 0.19	420.61 ± 31.94	4.36 ± 1.23
+iC&iN	25.06 ± 0.09	8.07 ± 0.05	6931.74 ± 712.58	1625.05 ± 129.18	45.21 ± 3.54	5682.74 ± 475.26	578.16 ± 122.35	8.97 ± 1.90	1676.55 ± 133.43	4.46 ± 1.09

## Supplementary Materials

The following are available online at https://www.mdpi.com/2076-2607/8/5/640/s1, Figure S1: Evolution of the ammonium uptake by *S. pistillata*, Figure S2: Evolution of the carotenoids cellular concentrations (peridinin + xanthophylls + β-carotene; ng cell^−1^) in the endosymbionts of the coral *S. pistillata*, Figure S3: Evolution of the chl-*a*/*c_2_* ratio in endosymbionts of the coral *S. pistillata*, Figure S4: Evolution of the maximal photochemical quantum yield (F_V_/F_M_) of *S. pistillata*, Figure S5: Evolution of the calcification rates of *S. pistillata*. Figure S6: rETR_PSII_ (μmol e^−^ m^−2^ s^−1^) and gross O_2_ evolution by PSII (E_O_; fmol O_2_ min^−1^ cell^−1^) at steady-state photosynthesis in *S. pistillata*,
