# Supplementary material for: Coral Productivity Is Co-Limited by Bicarbonate and Ammonium Availability
Source: Microorganisms. 2020 Apr 28;8(5):640. doi: 10.3390/microorganisms8050640 (PMC7285240; doi:10.3390/microorganisms8050640)
Supplement: Supplementary file 1 [file microorganisms-08-00640-s001.pdf]

Supporting information

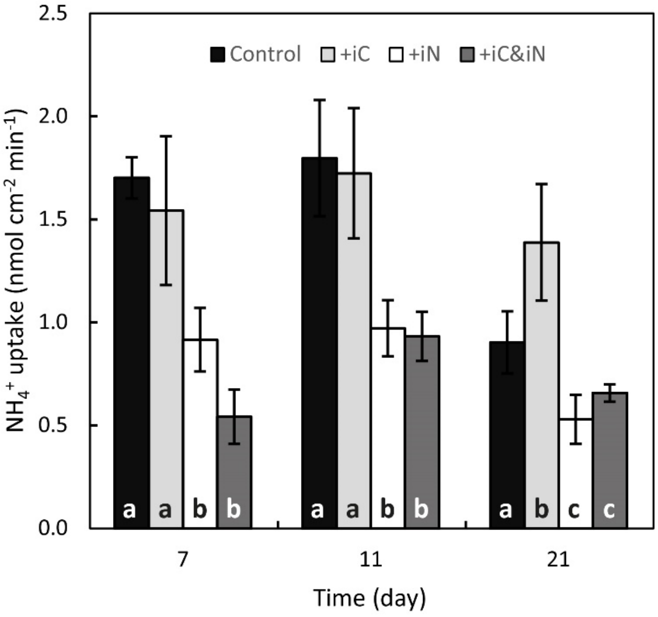

Figure S1. Evolution of the ammonium uptake by *S. pistillata* nubbins exposed for 3 weeks to natural seawater (control) and to seawater enriched with 6 mM HCO_3^- (+iC) or 4 µM NH_4^+ (+iN) or both (+iC & iN). Data are presented as mean ± SD (n = 6). Different letters indicate statistically significant differences between each treatment for one time point ($P < 0.05$).

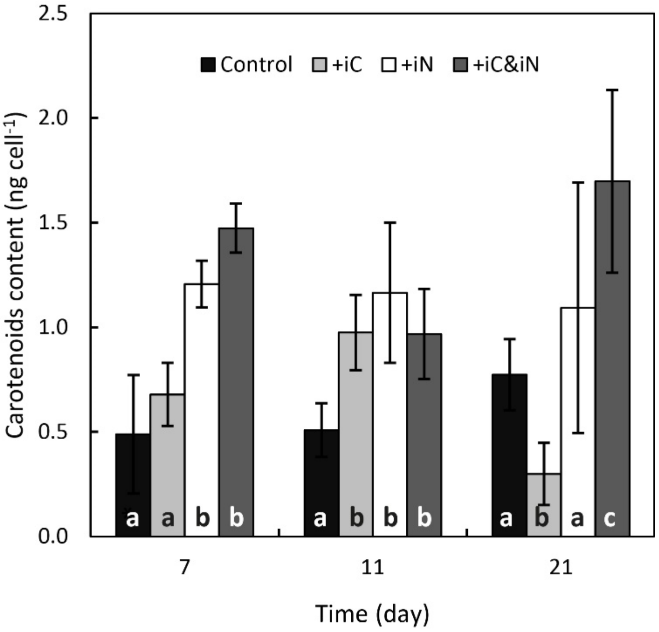

Figure S2. Evolution of the carotenoids cellular concentrations (peridinin + xanthophylls + β-carotene; ng cell^{-1}) in the endosymbionts of the coral *S. pistillata* exposed for 3 weeks to natural seawater (control) and to seawater enriched with 6 mM HCO_3^- (+iC) or 4 µM NH_4^+ (+iN) or both (+iC & iN). Data are presented as mean ± SD (n = 6). Different letters indicate statistically significant differences between each treatment for one time point ($P < 0.05$).

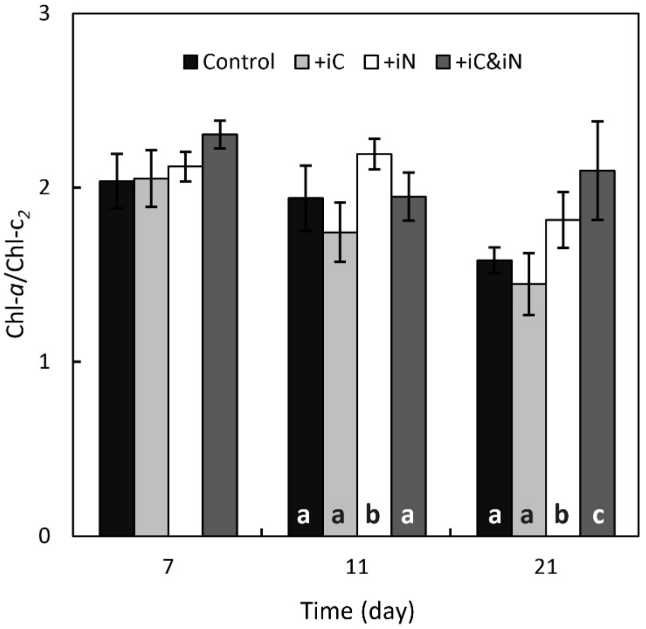

Figure S3. Evolution of the chl-*a*/*c*$_2$ ratio in endosymbionts of the coral *S. pistillata* exposed for 3 weeks to natural seawater (control) and to seawater enriched with 6 mM HCO_3^- (+iC) or 4 µM NH_4^+ (+iN) or both (+iC & iN). Data are presented as mean ± SD (n = 6). Different letters indicate statistically significant differences between each treatment for one time point (P < 0.05).

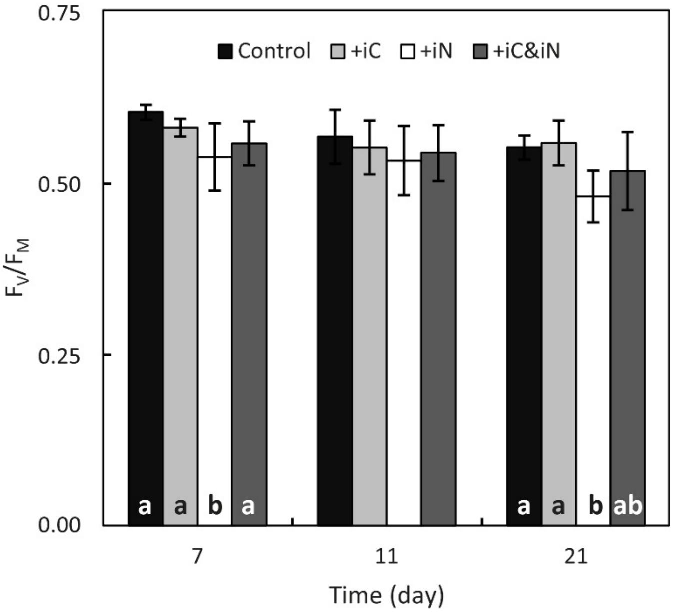

Figure S4. Evolution of the maximal photochemical quantum yield (F_V/F_M) of *S. pistillata* exposed for 3 weeks to natural seawater (control) and to seawater enriched with 6 mM HCO_3^- (+iC) or 4 µM NH_4^+ (+iN) or both (+iC & iN). Data are presented as mean ± SD (n = 6). Different letters indicate statistically significant differences between each treatment for one time point (P < 0.05).

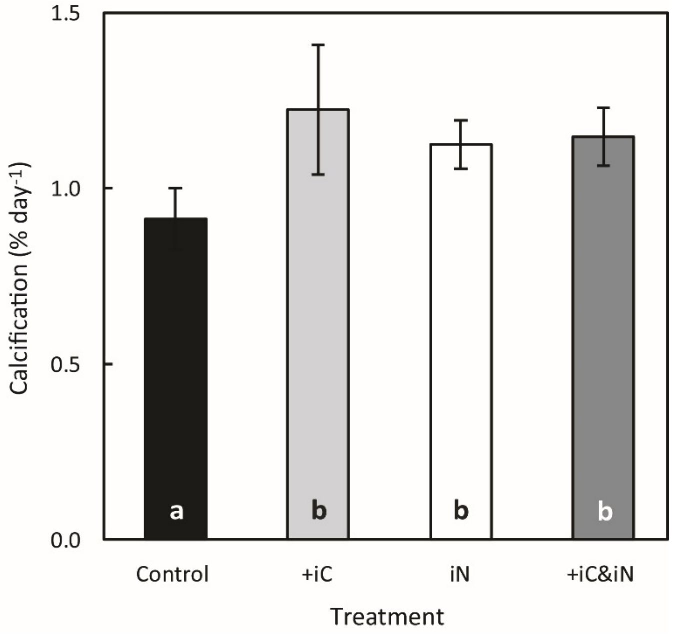

Figure S5. Evolution of the calcification rates of *S. pistillata* exposed for 3 weeks to natural seawater (control) and to seawater enriched with 6 mM HCO_3^- (+iC) or 4 µM NH_4^+ (+iN) or both (+iC & iN). Data are presented as mean ± SD (n = 6). Different letters indicate statistically significant differences between each treatment for one time point (P < 0.05).

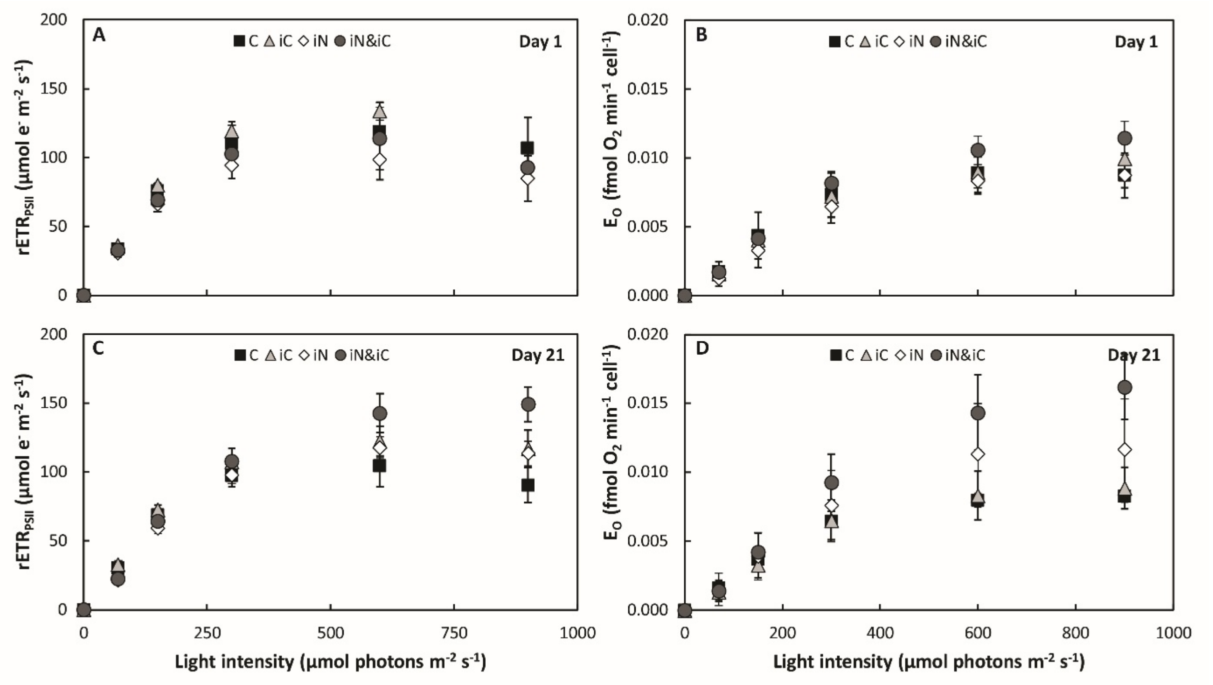

Figure S6. Relative electron transport rate through PSII ($rETR_{PSII}$; µmol e^- m^{-2} s^{-1}; A & C) and gross O_2 evolution by PSII (E_O; fmol O_2 min^{-1} $cell^{-1}$; B & D) at steady-state photosynthesis in *S. pistillata*, after 1 day and 3 weeks of exposure to natural seawater (control) and to seawater enriched with 6 mM HCO_3^- (+iC) or 4 µM NH_4^+ (+iN) or both (+iC & iN). Measurements were conducted at 0, 70, 150, 300, 600 and 900 µmol photons m^{-2} s^{-1} and data are presented as mean ± SD (n = 6).

Table S1. Mean values of the physiological parameters measured at the beginning of the experiment on *S. pistillata* nubbins (n = 24)

**Measured parameters**	**(units)**	**Mean ± SD**
***Symbiodinium* density**	(x 10^6 cells cm^{-2})	2.43 ± 0.21
**Eo***	(µmol O_2 min^{-1} cm^{-2})	0.023 ± 0.004
**Chlorophyll *a*+ *c2***	(µg cm^{-2})	6.61 ± 2.24
**Respiration**	(µmol O_2 s^{-1} cm^{-2})	-0.007 ± 0.001
**Chl-*a*/Chl-c_2**		2.04 ± 2.00
**F_V/F_M**		0.58 ± 0.03
**$rETR_{PSII}$***	(µmol e^- m^{-2} s^{-1})	116 ± 17
**NPQ***		2.94 ± 0.16
**O_2-dependent rETR**	(%)	0.75 ± 0.08

* These photosynthetic parameters were measured at a light intensity of 600 µmol photons m^{-2} s^{-1}
